# Genetic engineering strategies to enhance antitumor reactivity and reduce alloreactivity for allogeneic cell-based cancer therapy

**DOI:** 10.3389/fmed.2023.1135468

**Published:** 2023-03-29

**Authors:** Yuning Chen, Yichen Zhu, Adam Kramer, Ying Fang, Matthew Wilson, Yan-Ruide Li, Lili Yang

**Affiliations:** ^1^Department of Microbiology, Immunology & Molecular Genetics, University of California, Los Angeles, Los Angeles, CA, United States; ^2^Eli and Edythe Broad Center of Regenerative Medicine and Stem Cell Research, University of California, Los Angeles, Los Angeles, CA, United States; ^3^Jonsson Comprehensive Cancer Center, David Geffen School of Medicine, University of California, Los Angeles, Los Angeles, CA, United States; ^4^Molecular Biology Institute, University of California, Los Angeles, Los Angeles, CA, United States

**Keywords:** gene engineering, cancer immunotherapy, allogeneic cell products, CRISPR-Cas9, chimeric antigen receptor-engineered T (CAR-T) cells, immune checkpoints, graft-versus-host disease, alloreactivity

## Abstract

The realm of cell-based immunotherapy holds untapped potential for the development of next-generation cancer treatment through genetic engineering of chimeric antigen receptor (CAR)-engineered T (CAR-T) cell therapies for targeted eradication of cancerous malignancies. Such allogeneic “off-the-shelf” cell products can be advantageously manufactured in large quantities, stored for extended periods, and easily distributed to treat an exponential number of cancer patients. At current, patient risk of graft-versus-host disease (GvHD) and host-versus-graft (HvG) allorejection severely restrict the development of allogeneic CAR-T cell products. To address these limitations, a variety of genetic engineering strategies have been implemented to enhance antitumor efficacy, reduce GvHD and HvG onset, and improve the overall safety profile of T-cell based immunotherapies. In this review, we summarize these genetic engineering strategies and discuss the challenges and prospects these approaches provide to expedite progression of translational and clinical studies for adoption of a universal cell-based cancer immunotherapy.

## 1. Introduction

Recent developments in cell-based engineering have revolutionized the field of cancer treatment in terms of enhancing both the specificity and efficacy of potential therapeutics (1–3). Delivered agents such as immune checkpoint inhibitors or engineered immune cells bolster immunoreactivity and are designed to specifically target tumor cells with minimal off-target effects (Table 1) (2). Currently, cancer vaccines, oncolytic viruses, T and natural killer (NK) cells, stem cells, monoclonal antibodies and recombinant proteins are major areas of investigation for the development of new cancer therapeutics (3). As a relatively novel field of medicine, cell-based approaches provide a “living therapy” distinct from other forms of treatment, attracting massive interest as a highly malleable and dynamic platform (4, 5). Furthermore, compared to autologous cell therapy, wherein therapeutic cells are collected directly from the intended patient, manufactured and engineered *in vitro*, and used to exclusively treat that patient, allogeneic cell therapy can be preemptively positioned at treatment centers, manufactured at large scale, and is less logistically challenging and costly (6, 7).

**Table 1:** Engineering strategies for allogeneic cell products.

Engineering strategies	Target genes	Knock-out or overexpression	Outcomes	Reference
Enhance antitumor efficacy	CARs	Overexpression	Anti-tumor immunity enhancement; potential risk of GvHD and HvG effect	(5, 9, 29, 33)
	IL-15	Overexpression	T cell survival and persistence improvement; tumor-killing enhancement	(40, 41)
	IL-2	Overexpression	Tumor-killing enhancement; constitutive stimulation of immunosuppressive regulatory T cells; activation-induced cell death	(43)
	IL-7	Overexpression	Increased CD4^+^ and CD8^+^ T cells; improved tumor-targeting and killing	(44, 45)
	IL-12	Overexpression	Increased IFN-γ; greater antitumor efficacy; greater innate immune response enhancement	(47)
	IL-21	Overexpression	optimal proliferation, differentiation, and activation of T cells; greater cytotoxicity to tumor cells;	(43, 49–51)
	PD-1	Knock-out	PD-1&PD-L1/L2 interaction elimination; anti-tumor immunity enhancement	(53, 56, 58, 62–64)
	CTLA-4	Knock-out	CTLA-4&B7-1/2 interaction elimination; anti-tumor immunity enhancement	(53, 71)
	LAG-3	Knock-out	LAG-3&MHC interaction elimination; anti-tumor immunity enhancement	(73, 74)
	TIM-3	Knock-out	TIM-3&Galectin-9 interaction elimination; anti-tumor immunity enhancement	(76)
	NKG2A	Knock-out	NKG2A&HLA-E interaction elimination; anti-tumor immunity enhancement	(78, 84)
	EZH1	Knock-out	iPSC-derived T (EZ-T) cells production; robust anti-tumor responses;	(91–95)
	DNTM3A	Knock-out	HSC differentiation impairment amelioration; cell exhaustion prevention; anti-tumor activities improvement	(97–99)
	CD16	Overexpression	Better cell survival; anti-tumor activities improvement	(100–102, 104, 105)
Disrupt endogenous TCR to ameliorate GvHD	TCR	Knock-out	curtailment of graft cell capacity for immunoresponse; tumor recognition enhancement	(113, 114, 118–120)
	TCR	Engineered	Alloreaction amelioration; TCR-mediated antigen recognition retainment; antitumor capacity improvement	(122–124)
Reduce host cell-mediated allorejection	HLA	Knock-out	HvG effects abrogation; long-term graft persistence promotion	(6, 11, 136, 138, 143, 146)
	Inhibitory ligands	Overexpression	HvG effects abrogation; long-term tumor growth suppression enhancement	(150, 151, 153, 163, 164)
	CD52	Knock-out	total lymphodepletion avoidance; lower risk of GvHD	(165, 166)
Improve safety profile	Suicide genes	Overexpression	Selective destruction of modified cells; potential risks minimization	(2, 3, 5, 167)

Allogeneic hematopoietic stem cell transplantation (allo-HSCT) has been widely recognized as the earliest form of allogeneic cell therapy, after its implementation to treat inherited anemias and immune deficiencies in the 1950s (8). The Allo-HSCT platform was found to be particularly effective against hematological malignancies with limited response in solid tumors, and could potentially address refractory malignancies (8). Despite powerful graft-versus-tumor (GvT) implications, allo-HSCT-induced immune cell activation and proliferation also produced harmful graft-versus-host disease (GvHD) restricting its delivery.

Among peripheral lymphocytes, T and NK cells are the most commonly studied immune cells for antitumor allogeneic cell therapy, in the form of chimeric antigen receptor (CAR)-engineered T and NK (CAR-T and CAR-NK) cells (5, 9). Currently, all CAR-T cell therapies approved by the United States Food and Drug Administration (FDA) remain autologous, targeting CD19-positive B-cell leukemia and lymphoma (i.e., Kymriah, Yescarta, Tecartus, and Breyanzi), as well as B-cell maturation antigen (BCMA)-positive multiple myeloma (MM) (i.e., Abecma and Carvykti). Though CAR-T cells have shown less potency against solid tumors, several investigations are underway to target solid tumors with CARs. Ongoing clinical trials are targeted against several tumors, including prostate stem cell antigen (PSCA)-CAR-T cells against advanced prostate cancer, IL13Ra2-CAR T cells and epidermal growth factor receptor variant III (EGFRvIII)-CAR-T cells against advanced glioblastoma, glypican 3 (GPC3)-CAR-T cells against hepatocellular carcinoma (HCC), CD70-CAR-T cells against renal cell carcinoma (RCC), GD2-CAR-T cells against neuroblastoma, and mesothelin (MSLN)-CAR-T cells against MSLN-positive solid tumors (e.g., ovarian, breast, lung, and pancreatic cancer). However, these therapies are limited in their autologous nature due to potentially low healthy T cell numbers in patients, lengthy manufacturing process, and high cost. Allogeneic therapies address these limitations by increasing availability as an off-the-shelf therapy and reducing manufacturing variability, thereby decreasing the production costs.

Pioneering clinical trials are investigating allogeneic cell therapy by collecting peripheral blood mononuclear cells (PBMCs) *via* leukapheresis from healthy donors for *in vitro* expansion (9, 10). Further engineering involves arming of these cells with tumor antigen-specific CARs and ablation of the TRAC locus to eliminate the risk of GvHD (9, 11, 12). Some trials also involve engineering cord blood-derived hematopoietic stem cells (HSCs) or induced pluripotent stem cells (iPSCs) to generate NK cells with CARs enhancing NK-mediated killing (9). The resulting genome-edited, donor-derived allogeneic CAR-T cell products can be distributed to treat multiple patients (10, 12). In addition to conventional T or NK cells, innate-like T cell subtypes including invariant natural killer T (iNKT) and mucosal-associated invariant T (MAIT) cells have been explored due to their limited TCR repertoires that evade MHC restriction, suggesting their potential as a highly efficient allogeneic cell product (10, 11).

Several sources have been investigated for the generation of allogeneic cell therapies, including PBMCs, iPSCs, and HSCs. PBMCs are the simplest cell product to derive by isolating the desired cell type from healthy donors *via* leukapheresis, engineering *via* retro-or lentiviral transduction, and culturing to high purity. However, stem cell-derived therapies provide a more beneficial and malleable platform for therapeutic cell production (13). These approaches, including HSC-and iPSC-differentiation, hold several advantages over PBMC-derived cell generation; ubiquitously, engineering nascent stem cells has lower cost and produces higher yields of therapeutic cells (13, 14). iPSCs can be derived from any mature cells, commonly skin cells, and can be differentiated into any cell type. HSCs are derived from bone marrow or cord blood donors and can be differentiated into any hematopoietic lineage cell including blood cells, immune cells, or platelets. Current pre-clinical and clinical research has focused on HSCs and iPSCs as the initial source for engineered allogeneic cell products. We have demonstrated that human CD34^+^ cord blood-derived HSCs could be cultured, engineered, and differentiated into CAR-engineered iNKT (CAR-iNKT) cells with high yield and purity. These cells show high tumor-killing specificity and efficacy in murine models (6, 15, 16). In addition, current research involving iPSCs show that these cells can be guided across human leukocyte antigen (HLA) barriers to differentiate into many therapeutic cell types including CD8 T cells and NK cells (17–19). Clinical trials for iPSC-derived CAR-NK cells show promising results against hematological and solid malignancies in humans (19). Thus, drawbacks in manufacturing throughput and efficacy characteristic of primary immune cell therapies could be overcome through incorporation of stem cells (6, 19).

Nevertheless, allogeneic cell-based cancer immunotherapy still has several noteworthy limitations. Most notably, graft immunosuppression challenges both off-the-shelf cell products and autologous cell-based therapies alike. For instance, the heterogeneous tumor microenvironment (TME) associated with solid tumors suppresses effector cell activation and antitumor capacity through anti-inflammatory agents including tumor-associated macrophages (TAMs), myeloid-derived suppressor cells (MDSCs), and anti-inflammatory cytokines (9, 20). Additionally, allogeneic cell products typically induce dual GvHD and host-versus-graft (HvG) effects from immunogenic HLA mismatch between donor and recipient (10, 19). TCR recognition of effector cells against healthy host cells by donor effector cells unleashes cytotoxic cascade through expression of FAS ligand, perforin, and granzymes (10). iPSC-dervied NK cells have demonstrated the capability to produce a hyperinflammatory environment, inducing cytokine release syndrome (CRS) in recipients (21). Additionally, HvG effect in host rejection of graft cells reduces the antitumor efficacy and persistence of allogeneic cells, precluding maximum therapeutic capacity (11).

To address these problems, diverse approaches have been explored and implemented in cancer research, leading to significant advancement in effector cell capabilities. The invention of genetic engineering techniques such as viral transduction, CRISPR-Cas9, and transcription activator-like effector nuclease (TALEN) largely expands the potential to artificially modulate genetic expression of therapeutic cells. In this review, we discuss the major obstacles that hinder cancer treatment and summarize current genetic engineering strategies to overcome these difficulties (Table 1). Enhancement of antitumor efficacy, amelioration of GvHD, reduction of HvG effect, and improvement of safety profiles are essential directions for the progression and adoption of next generation of allogeneic cell-based cancer immunotherapy.

## 2. Engineering strategies to enhance antitumor efficacy

### 2.1. Engineering of chimeric antigen receptors (CARs)

CARs are an engineering milestone in synthetic biology responsible for unprecedented response rates in patients since its initial U.S. FDA approval in 2017 for CD19-targeting CAR-T cell therapy against relapsed or refractory B cell acute lymphoblastic leukemia (ALL) (22). CARs were first developed in T cells with four distinct functional domains: an extracellular antigen-recognition domain, a flexible hinge region, a transmembrane domain, and an intracellular T cell signaling domain (23). The extracellular antigen-binding domain could be comprised of various structures including single chain variable fragments of antibodies (scFv) (24), cell receptors (25), ligands (26), other derived peptides programmed for specific interactions (27), or nanobodies (28). This domain is constructed with a range of specificities to the specified target to provide ample stimulation while avoiding activation-induced cell death (23). Several studied targets for the extracellular domain in hematological malignancies include, but are not limited to, CD19, CD20, CD22, CD30, CD38, and BCMA. Targets in solid tumors include human epidermal growth factor 2 (HER2), epidermal growth factor receptor (EGFR), GD2, mucin 1, mesothelin, programmed death ligand 1 (PD-L1), and CD171, among others (29).

The hinge region connects the extracellular domain to the transmembrane domain, which then connects the structure to the intracellular signaling domain. First-generation CARs are composed of this basic structure, with the intracellular domain typically a CD3ζ domain for T cell activation (30). Second-and third-generation CARs consist of one or two additional costimulatory domains, respectively; most commonly, 4-1BB or CD28 costimulatory domains are can included to provide enhanced activation and expansion (31). Fourth-generation CARs include a cytokine-releasing cassette that is constitutive or induced, allowing for increased toxicity or proliferation by cytokine stimulation (e.g., IL-12, IL-15, and IL-18) (31, 32).

CARs are most commonly engineered on T cells harvested from the patient receiving treatment. So far, patient-derived T cells are the base of each FDA-approved CAR-T cell therapy, with allogeneic T cells posing the risk of graft-versus-host alloreaction due to histoincompatibility (29). CAR-T cells also pose increased risk due to cytokine release syndrome (CRS) and neurotoxicity (33). Alternative cells for CAR-engineering include NK (34) and unconventional innate T cells, such as iNKT, MAIT and gamma delta T (γδ T) cells (14). These alternative effector cells demonstrate reduced risk of allorejection and exhibit potential for off-the-shelf cell products derived from healthy donor PBMCs and stem cells (14). Furthermore, they exhibit a shorter duration of activity and cause less severe toxicities, while also utilizing multiple cytotoxic mechanisms that are not dependent on CAR signaling to enhance tumor targeting (35, 36).

One popular extracellular domain for CAR-NK cells is NKG2D, an NK activating receptor that recognizes stress-induced ligands including MICA, MICB, and ULBP that are expressed on DNA-damaged or transformed cells (37). NKG2D-CAR-NK cells exhibit enhanced killing and higher specificity than conventional NK cells, with the ability to augment persistence with IL-15 co-expression (37, 38). Furthermore, compared to CAR-T cells, NKG2D-CAR-NK cells exhibit lower expression of cytotoxic molecules such as granzyme B, perforin, and IFN-γ while showing better tumor-killing and survival *in vivo*, attributed to an increase in the expression of immunological activation genes (37). iNKT cells also exhibit benefits in CAR-engineering against hematological malignancies, targeting acute myeloid leukemia and B cell lymphoma more efficiently than CAR-T cells while ameliorating GvHD from allogeneic transplantation (14). GvHD ablation by iNKT cells was shown to be achieved by the rapid depletion of CD14^+^ myeloid cells responsible for exacerbated alloreaction in mice (14).

### 2.2. Incorporation of cytokine armoring genes

One barrier restricting cell-based cancer therapy is limited efficacy due to the downregulation of costimulatory molecules on tumor cells (39). The genes responsible for producing cytokines, which are crucial for the proper function and persistence of immune cells, have been modified and incorporated into engineered cells, resulting in an improved ability of these cells to specifically target tumors. IL-15 is an important cytokine in CD8^+^ T cell and NK cell stimulation and proliferation (40). It has been demonstrated the engineered ectopic expression of IL-15 in CD19-CAR-T cells results in 3-to 15-fold greater expansion, survival, antitumor efficacy, and decreased PD-1 expression *in vivo* (41). Membrane-bound IL-15 engineered on CD19-CAR-T with a platform to promote T-memory stem cells (T_SCM_) showed similar effects, increasing persistence *in vivo* and promoting a CD45RO^neg^CCR7^+^CD95^+^ phenotype akin to T_SCM_. T-cell persistence independent of CAR signaling was achieved by the membrane-bound IL-15 due to STAT5 signaling, enabling CAR-T cells to undergo long-term memory stem-cell differentiation (42). IL-2 has been studied as a key stimulant of T cells similar to IL-15, however its constitutive stimulation of immunosuppressive regulatory T cells limits its effectiveness in cancer therapies. IL-2 is also associated with activation-induced cell death, limiting long-term activation and durability *in vivo* (43).

IL-7 is an important cytokine for lymphoid differentiation from hematopoietic stem cells. Administration of IL-7 exhibits increases in CD4^+^ and CD8^+^ T cells in circulation dependent on dose (44). In comparison to IL-15, IL-7 promotes the expansion of naïve T cells and leads to a more potent tumor-killing response (44). Transgenic IL-7 expression in CAR-T cells has therefore been explored and proven to increase tumor-targeting and killing in the presence of regulatory T cells in hematological and solid tumors (45). Specifically, CD20-, CD19-, GD2-, and mesothelin-CAR-T cells showed enhanced proliferation and cytotoxicity. These were much improved with the co-expression of CCL19 or CCL21 to further improve chemotaxis and proliferation (45, 46).

IL-12 is associated with a pro-inflammatory immune response in both innate and adaptive immune cells. Genetically modifying CAR-T cells to express IL-12 upon CAR or TCR stimulation under the control of the NFAT promoter provides T cells with a steady and high level of IL-12 secretion. By controlling IL-12 expression with an NFAT response, IL-12 is restricted in tissues the effector cells pass through, limiting toxicity in healthy tissues (47). In tumor tissues IL-12-engineered CAR-T cells exhibited elevated IFN-γ secretion, greater antitumor efficacy, and greater innate immune response (47). By activating both the innate and adaptive immune response, IL-12 may enhance tumor-targeting against cells that have undergone antigen-escape to avoid killing by antigen-specific CAR-T cells. Innate response is evidenced by elevated levels of macrophages, NK, and NKp46^+^ cells following IL-12 expression within the tumor environment (48). Studies have also shown that IL-12 increases antigen processing and presentation to increase T cell stimulation indirectly (49). Engineering inducible IL-12 activation is being investigated in phase I/II clinical trials for an EGFR-specific CAR-T cell against metastatic colorectal cancer (43).

IL-21 is an essential cytokine for optimal proliferation, differentiation, and activation of T cells utilizing the STAT3 pathway (49). Batra et al. demonstrated that GPC3-CAR-T cells coexpressing IL-21 effectively target GPC3-positive tumor lines, with the most robust expansion and persistence in cells expressing combined IL-21 and IL-15 (50). IL-21-expressing cells exhibited greater cytotoxicity to tumor cells and greater survival in mice than IL-15 or IL-2 treated CAR-T cells despite higher levels of IFN-γ and Bcl-2 expression (43, 50, 51). Because the levels of circulating IL-15 and IL-21 in mouse peripheral blood were 100-1,000-fold lower than the maximum tolerable dose in humans, systemic toxicities are not anticipated with CAR-T cells coexpressing these cytokines (50, 51). The efficacy of these engineered T cells are being evaluated in two ongoing clinical trials (NCT02932956 and NCT02905188) for GPC3^+^ liver cancer and hepatocellular carcinoma (52).

### 2.3. Blockade of immune checkpoint receptors

**Figure 1:**
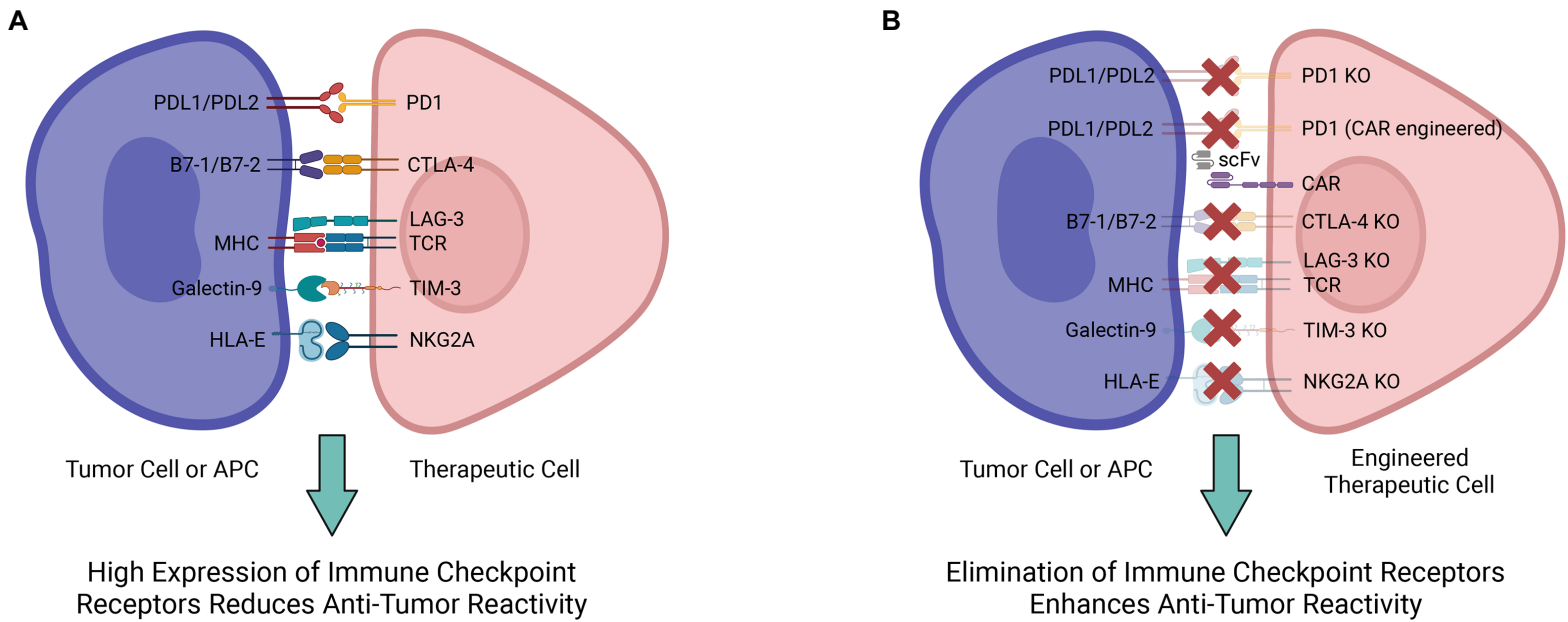
Multiple engineering strategies to mitigate the immune checkpoint receptor-mediated immunosuppression. **(A)** Various ligand-receptor interactions are involved in the immunosuppression and decrease antitumor reactivity of therapeutic cells. **(B)** Engineering therapeutic cells to ablate their immune checkpoint receptors and interrupt ligand-receptor interactions for enhanced antitumor reactivity.

Immune checkpoint blockade has rapidly become one of the most promising therapeutic strategies for triggering anti-tumor immunity. As a key component of the immune system, immune checkpoints are essential for preserving self-tolerance as well as regulating the intensity and duration of physiological immune response in peripheral tissues to protect cells from being attacked indiscriminately (53). Tumor cells are able to express immune checkpoint ligands interacting with receptors on immune cells to inhibit their activity, and thereby bypass the immune system. This has limited cell therapies by enabling tumor cells to mask themselves from immune cells, causing immune resistance. To address the immune suppression against tumors from this ligand-receptor interaction, antibodies for immune checkpoint blockade have shown promise in blocking the interaction to enable tumor-targeting by immune cells (Figure 1).

Among the diverse immune checkpoint proteins, two of the most extensively studied are programmed cell death protein 1 (PD-1) and cytotoxic T-lymphocyte-associated protein 4 (CTLA-4) (54). Generally, PD-1 is responsible for maintaining peripheral tolerance during cancer, infection, or chronic inflammation (54, 55). PD-1 ligands PD-L1 and PD-L2 expressed in tumor cells bind to PD-1 expressed on the surface of T cells, leading to inhibitory checkpoint signaling that reduces cytotoxicity and T-cell exhaustion (56). The PD-1/PD-L1/2 interaction downregulates the immune responses and provides an opportunity for tumor cells to persist uninterrupted. In response to this immune resistance, anti-PD-1 treatment is aimed at enhancing T cell function by blocking the inhibitory signals (53). By identifying mutated peptides, such as neoantigens, tumor-infiltrating T cells can trigger a potent immune response (57). Specifically, nivolumab, an FDA-approved antibody targeting human PD-1, has shown clinical efficacy in 2010 and 2012 against mesothelioma and gastric cancer (58, 59). According to a phase I clinical study of nivolumab, treatment efficacy was correlated with the expression of PD-1 ligands in tumor cells (58). However, PD-1 ligands are differentially expressed across different tumors; it is predicted that the therapeutic effect is selective for PD-1^high^ tumors (e.g., lung cancer, glioblastoma, melanoma) (55, 60).

CARs also provide a potent mechanism for PD-1 blockade. Because of the immunosuppressive TME and feeble natural immune responses against tumors, clinical trials utilizing checkpoint inhibitors have shown limited effectiveness regarding some types of tumors, such as pancreatic ductal adenocarcinoma (61, 62). Despite the promise of PD-1 blockadeas a form of cancer therapy, its efficacy may be limited for certain types of cancer and between patients due to tumor heterogeneity. CARs can disrupt the interaction between PD-1 and PD-L1 to effectively treat PD-L1^+^ cancers. For example, in pancreatic cancer (PaC) third-generation CARs with costimulatory domains such as CD28, CD137, and OX40, can effectively target PD-L1 on PaC cells. Furthermore, in xenograft/orthotopic mouse models, the targeting specificity of CARs largely determines the cytotoxic activity of the engineered CAR-T cells, with greater binding affinity of the scFv associated with greater cytotoxicity (62–64).

In contrast to PD-1, the CTLA-4 immune checkpoint regulates T-cell proliferation in lymph nodes during the early stages of an immune response (65). CTLA-4 maintains immune balance by downregulating the function of activated T cells through inhibitory signals from binding B7, a membrane protein expressed on activated antigen presenting cells (APCs) (65). Anti-CTLA-4 monoclonal antibodies such as ipilimumab bind CTLA-4 to shut down inhibitory signals, enabling CTLs to persist with their cytotoxic activities against cancer cells (53). Furthermore, preclinical data has indicated that CTLA-4-blockade has a significant effect on boosting the anti-tumor response, suggesting that targeting CTLA-4 could serve as a viable method for activating cytotoxic T-lymphocytes (CTLs) in cancer therapy (66–68). In metastatic melanoma, ipilimumab improved patients’ survival after established lines of treatments had failed (69, 70). CRISPR-Cas9 has been utilized to target CTLA-4 as well. To construct CTLA-4 knockout CTLs, lentiviral vectors containing guide RNA specific for CTLA-4 are used to create double-strand breaks and block CTLA-4 expression (71). Following CTLA-4 knockout, anti-tumor cytotoxicity and cytokine secretion by CTLs are notably increased, as well as the apoptosis and caspase activities of tumor cells. This is indicative of an overall improvement in antitumor efficacy following CTLA-4 knockout with CRISPR-Cas9 (71).

In addition to PD-1 and CTLA-4, several new immunological checkpoints have been discovered. Lymphocyte activation gene-3 (LAG-3), adjacent to the CD4 gene on chromosome 12, is a negative regulator for T cell activities (54). Structural similarities between LAG-3 and CD4 make MHC-II a high-affinity ligand of LAG-3 (72). CRISPR-Cas9 is also employed to understand the mechanisms behind LAG-3 checkpoint regulation. After using CRISPR-Cas9 with electroporation to knockout LAG-3 in both T cells and CAR-T cells, the immune phenotype and viability of the edited cells are not significantly different from the control cells *in vitro*. However, *in vivo* studies demonstrate that LAG-3 modulates pool size and downregulates T cell expansion, evident from increased T cell populations in LAG-3 knockout models (73, 74). Though further research is required to understand the functions of LAG-3, gene-editing provides a potential mechanism for enhancing lymphocyte recognition of tumor cells.

T cell immunoglobulin mucin family member 3 (TIM-3), also known as hepatitis A virus cellular receptor 2 (HAVCR2), has been increasingly studied because of its distinct characteristics as a suppressive marker. As a type I transmembrane protein, TIM-3 is essential for suppressing Th1 responses and the production of cytokines, including TNF and INF-γ. TIM-3-associated dysregulation is usually implicated in autoimmune disease. Generally, Tim-3 expression on T cells is inversely linked with the progression of autoimmune disorders (75). Furthermore, as the TIM-3 expression level increases, the level of T cell exhaustion increases as well, marking the gradual loss of T cell function during long-term viral infections and tumor growth. Studies on both human and mouse tumors indicate that TIM-3 controls T cell exhaustion in tumor-infiltrating leukocytes (TILs), acting as a checkpoint for tumor immunity (75). High TIM-3 expression on CD8^+^ T cells has been correlated with poor prognosis in cancer patients. Moreover, TIM-3 and PD-1 expression on CD8^+^ TILs are highly correlated (76). With increased research in TIM-3 and CRISPR knockouts, evidence is mounting that double-positive TIM-3^+^PD-1^+^ CD8^+^ T cells are linked to higher levels of T cell exhaustion (77). Further research utilizing TIM-3 knockouts will be able to uncover the mechanisms behind TIM3-mediated suppression and may evidence greater potential for therapeutics.

NKG2A, an inhibitory regulator in the NKG2 family of proteins, is an important immune checkpoint for both CD8^+^ T cells and NK cells. Similar to other immune checkpoints, NKG2A binds to its ligand, peptide-presenting human leukocyte antigen-E (HLA-E), to activate inhibitory signals (78). The expression pattern of NKG2A in CD8^+^ T cells is highly regulated. In healthy individuals, NKG2A expression is minimal in CD8^+^ T cells, whereas during tumor development and persistent viral infection, the expression level increases (79, 80). Cytokines (e.g., IL-2, IL-4, and IL-6) can effectively modulate NKG2A expression on CD8^+^ T cells. NKG2/CD94 receptor interaction conducts inhibitory signals in CD8^+^ T cells, leading to a decrease in cytotoxic activity (81). NKG2A is present in about half of the human peripheral blood NK cells. Although NKG2/CD94 activation has a restrictive effect on the cytotoxicity of NK cells, interrupting the receptor-ligand interaction with HLA-E reinvigorates NK killing (78). Tumors often overexpress HLA-E and are thus able to suppress NK antitumor activity. In fact, HLA-E overexpression is associated with poor prognoses in several cancers, including glioblastoma and breast cancer (82, 83). NKG2A-mediated immune suppression in NK cells has been alleviated *via* NKG2A protein expression blocker (PEBL) transduction and anti-NKG2A antibody interference. PEBL transduction has been shown to induce a higher level of NK cell cytotoxicity without risking the relapse of NKG2A expression (84). Based on data from murine models, downregulating NKG2A effectively enhances anti-tumor immune response in both CD8^+^ T cells and NK cells (84).

Antigen spreading, a result of sequential immune response, must be taken into consideration during immune checkpoint blockade (85). This phenomenon involves broadening the immune response from the initial targeted response against a particular epitope to include other subdominant or previously unrecognized epitopes. Such a process may occur in response to self and foreign peptides, and it can lead to a more diverse an `11d robust immune response against the target antigen (86). Although past clinical trials did not investigate antigen spreading in immune checkpoint inhibition, there have been reports demonstrating that patients responding to the treatment experienced the recruitment and expansion of tumor-specific T cells that were not detectable before the therapy (87–90). Additionally, there has been experimental evidence indicating that checkpoint inhibition therapy was able to induce antigen spreading (85). Further studies are needed to analyze how the occurrence of antigen spreading could enhance the efficiency of immune checkpoint inhibition therapy.

### 2.4. Modulation of transcription factors and epigenetic enzymes

Transcription factors and epigenetic enzyme modulation play vital roles in immune cell activation, differentiation, and effector function (91). One frequently studied enzyme is the histone methyltransferase EZH1, an antagonistic lymphoid potential regulator during embryonic hematopoiesis. Human induced pluripotent stem cells (iPSCs) are essential resources for cell therapies, but deriving mature cell types can be difficult (92). Studies have shown that using iPSCs derived from T cells that have productively altered TCRs or incorporating organoid or thymic culture systems results in improved T cell maturation *in vitro* (93–95). To further improve the outcomes, EZH1 programming is included during cell derivation. By coupling a stroma-free T cell differentiation system with EZH1-knockdown-mediated epigenetic engineering, iPSC-derived T (EZ-T) cells can be produced. These artificially generated cells possess mature molecular markers similar to those found on peripheral blood-derived TCRαβ T cells and exhibit a very diverse TCR repertoire (92). Effector and memory T cell subsets are produced as a result of EZ-T cell activation. EZ-T cells display robust anti-tumor responses both *in vitro* and *in vivo* in xenograft models after being transduced with CARs. Developmentally mature T cells can be efficiently produced from iPSCs and employed in adoptive cell therapy due to epigenetic remodeling of EZH1 repression (92).

Another crucial reprogramming method for increasing anti-tumor efficacy is *via* DNA methyltransferase 3A (DNMT3A), an enzyme that catalyzes 5-methylcytosine methylation (96). DNMT3A is an essential factor in both embryonic and hematopoietic stem cell (HSC) differentiation (96). The loss of DNMT3A causes long-term HSC differentiation impairment *in vivo* (97). Mutations of DNMT3A can be found in human HSCs and are often associated with hematological malignancies (96). Due to this characteristic of DNMT3A, reprogramming has become a research target and therapeutic method for both hematological and solid malignancies. Epigenetically altered CAR-T cells with modulated DNMT3A have been studied to further understand the role of DNMT3A in anti-tumor activities. One significant drawback of traditional CAR-T cell therapy is exhaustion during prolonged antigen exposure. Under antigen stimulation, the expression of DNMT3A is upregulated by T cells. While the DNMT3A-regulated epigenetic changes in CAR-T cells induce exhaustion, removing DNMT3A from CAR-T cells improves the anti-tumor activities and avoids possible dysfunction in an IL-10-dependent manner (98). CRISPR-Cas9 can be used to generate CAR-T cells with DNMT3A knocked out with consequent constant proliferation and successful control of tumor development *in vivo,* exhibiting the benefits of targeting DNMT3A in immunotherapies (99).

### 2.5. Induction of CD16 to mediate antibody-dependent cellular cytotoxicity (ADCC)

One mechanism ADCC is mediated is through immunoglobulin G (IgG) Fc receptor FcγRIIIa, otherwise known as CD16a. On NK cells CD16a is associated with CD3ζ or Fcɛ receptor I (FcɛRI) γ chains. When it binds to the Fc portion of IgG, CD16a causes the cell to undergo biochemical events similar to T cell activation and lead to cytotoxicity (100, 101). Following NK cell activation, a disintegrin and metalloproteinase-17 (ADAM17) cleaves CD16a, reducing ADCC capacity and leading to NK cell dysfunction (101). FDA-approved monoclonal antibodies such as rituximab (anti-CD20) and trastuzumab (anti-CD19) are aimed at bringing NK cells within proximity of tumor cells and activating ADCC function to kill the tumor (102).

Zhu et al. have engineered “off-the-shelf” NK cells from induced pluripotent stem cells (iPSC) and engineered high-affinity non-cleavable CD16a (hnCD16a) to maintain NK cytotoxicity (102). These cells expressed similar NK activation and maturation markers but showed much higher CD16a expression and persistence. This was achieved by replacing a serine residue with a proline in the membrane proximal region of the CD16 receptor (103). *In vitro* and *in vivo* assays demonstrate hnCD16a-NK cells with a single dose of monoclonal antibody mediate ADCC against lymphoma and ovarian cancer, with better survival than PBMC-NK cells (102). Engineered hnCD16-NK cells from iPSCs serve as a potential allogeneic cell therapy because of the lack of HLA specificity in NK cells. Cichocki et al. (104) reported engineered CD16-CAR-NK cells with membrane-bound IL-15 from iPSCs capable of targeting multiple myeloma and being mass produced. These data showed enhanced durability without supplemention with stimulatiory cytokines against NSG models of multiple myeloma. Because of their potential for adoptive transfer without inducing GvHD, several clinical trials are in progress to induce ADCC against B cell lymphoma, pancreatic and other advanced solid tumors, and multiple myeloma *via* monoclonal antibody/CD16-NK cell combined treatment (105).

## 3. Engineering strategies to ablate endogenous TCR expression

Expression of HLA molecules largely mediates the clinical manifestation of GvHD for adoptive cell therapy recipients on account of alloreactivity against host-mismatch recognition. Prior studies have shown the capacity to suppress GvHD while maintaining the desired GvL reaction through depletion of mature alloreactive graft cells (106–109), dosage titration (110), and allo-anergization through exposure to host cells *ex vivo* (111, 112). The standardization of genetic editing techniques has since drawn attention towards the viability of employing genome-based methods to preclude GvHD onset. In particular, disrupting expression of a functional TCR at the constituent TCR receptor α constant (TRAC) and β constant (TRBC) loci effectively curtails graft cell capacity for immunoresponse, including alloreaction (113, 114). In order to produce a viable graft, insertion of a chimeric antigen receptor (CAR) gene at the TRAC/TRBC loci can effectively substitute a functional TCR. A wide variety of differentially expressed tumor-associated antigens have been identified within malignancies (e.g., BCMA, CD19, GD2, and Mesothelin) which can be selectively targeted by the CAR depending on tumor expression profile (115–117). Clinical trials administering CD19-targeting CAR-T cell therapy that lacked endogenous TCR demmonstrated enhanced tumor recognition despite its missing receptor, suggesting the feasibility of adopting a universal CAR-T therapy for cancer treatment (118–120).

However, limitations including antigen escape and graft impurity limit the safety of adopting a “universal” CAR-T cell-based therapy (121). Other promising methods employ a transgene encoding a non-conventional TCR that does not engage in alloreactivity while retaining TCR-mediated antigen recognition; so far this strategy has been most prominently adopted using NY-ESO-1 TCR transgene. The NY-ESO-1 TCR displays enhanced affinity for the antigen NY-ESO-1 when complexed with HLA-A2, both of which are highly expressed on the surface of multiple myeloma MM and testicular cancer cells (122, 123). A study performed by Mastaglio et al. employed zinc finger nuclease (ZFN) to disrupt the TRAC locus with a copy of the NY-ESO-1 TCR gene produce NY-ESO-1 T cells; compared to a non-TCR knockout graft, NY-ESO-1 T cells maintained similar reduction of NY-ESO-1^+^ MM tumor burden while abating GvHD response (124). Single TRAC knockout was sufficient to abolish endogenous TCR formation and suppress alloreactivity, attributed to non-productive mispairing of the remaining TCR β chain with NY-ESO-1 TCR α chain.

Another viable alternative lies in using unconventional T cell subsets, such as iNKT cells, that recognize antigens *via* an invariant TCR, independent of HLA. The human iNKT TCR is composed of a Vα24-Jα17 chain paired with a heavily restricted repertoire of Vβ chains, primarily Vβ11 (125, 126). Unlike conventional TCRs, the iNKT TCR recognizes antigens in an HLA-independent manner, restricted to glycolipids presented by CD1d, a non-classical MHC molecule (127). By virtue of their inability to recognize HLA antigen complexes, iNKT cells possess powerful antitumor capacity without the risk of inducing GvHD response (6, 128). Additionally, iNKT expression of NK activating receptors (i.e., DNAM-1, NKG2D, NKp33) permit alternative, TCR-independent recognition of tumors (129, 130). Clinical engraftment of iNKT cells against a variety of malignancies, including MM (130), lung cancer (131), and head and neck cancer (132), demonstrated significant reduction in tumor burden with greater safety profile compared to checkpoint inhibitor and CAR-T cell therapies. The improved safety of iNKT cell adoptive transfer, as well as its dual employment of innate and adaptive immune response position iNKT cells as a prime candidate for allogeneic “off-the-shelf” cancer immunotherapy.

## 4. Engineering strategies to reduce host-versus-graft (HvG) effect

### 4.1. Ablation of HLAs

**Figure 2:**
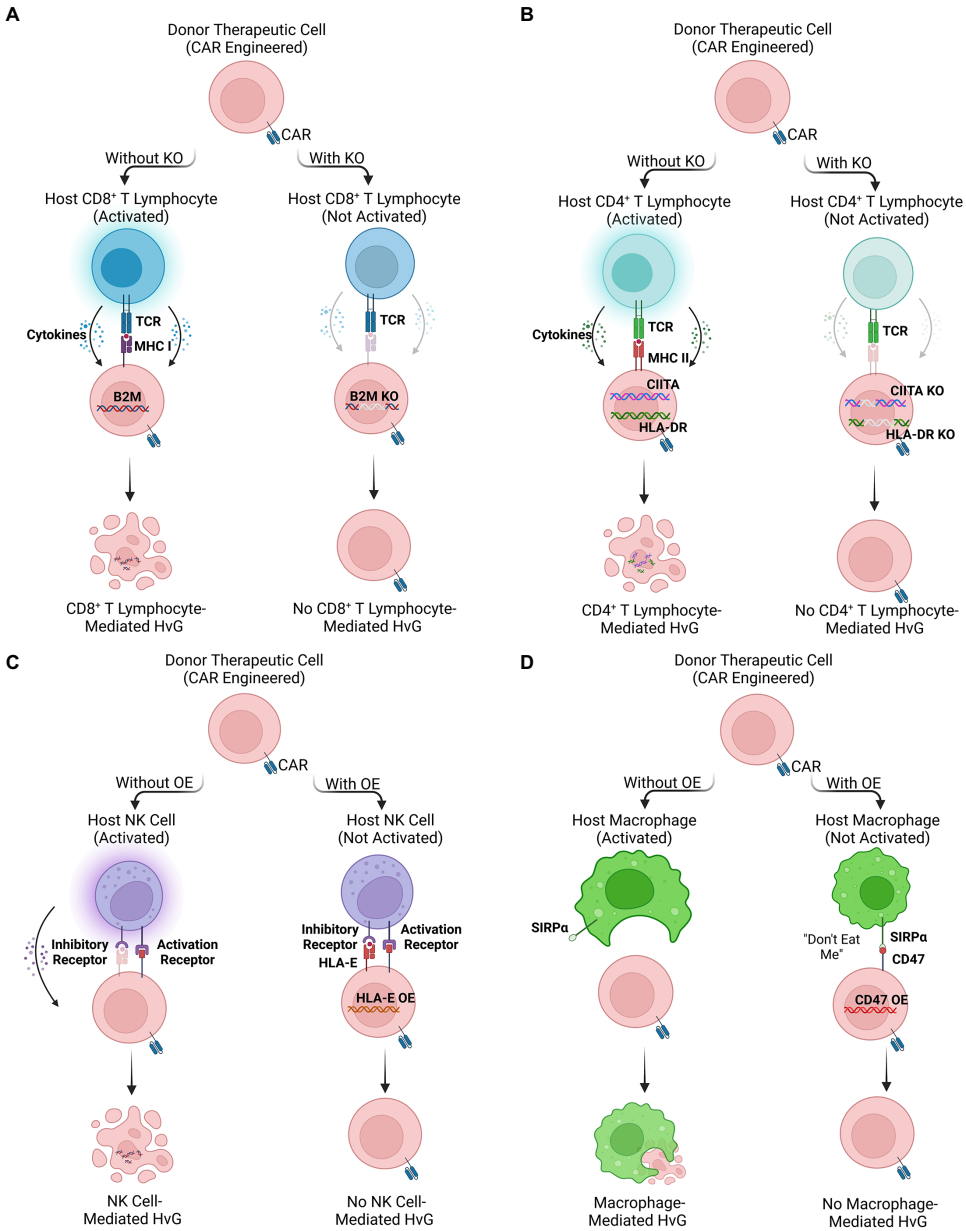
Multiple engineering strategies to reduce host-versus-graft (HvG) effect. **(A,B)** The ablation of HLA molecules expressed on therapeutic cells by knocking out HLA-related genes (e.g., B2M, HLA-DR, and CIITA), avoiding CD8^+^ and CD4^+^ T lymphocyte-mediated HvG effect. **(C,D)** The overexpression of inhibitory ligands (e.g., HLA-E and CD47) of therapeutic cells. The binding of inhibitory ligands prevents NK signaling pathway and macrophage phagocytosis, reducing NK cell-and macrophage-mediated HvG effect.

Beta-2 microglobulin (B2M) monomers form a functional complex with major histocompatibility class I (MHC I) molecules that are universally expressed on the surface of nucleated cells (133). The mature MHC I complex organizes presentation of intracellular antigens towards its cognate CD8 co-receptor expressed by single positive CD8 cytotoxic T cells. Cytosolic proteins are degraded into antigen peptides and loaded onto MHC Class I molecule (134). This complex translocates to the cell surface for TCR-mediated recognition of loaded antigens, inducing activation of CD8^+^ cytotoxic lymphocytes (Figure 2A). Expression of MHC I restricts the implementation of allogeneic therapy twofold. First, MHC I mismatch on graft cells activates endogenous lymphocytes producing a HvG effect that limits the survival and persistence of therapeutic donor cells (135). Secondly, cell therapies that function using TCR-mediated pathways recognize MHC I mismatch on host cells and induce onset of GvHD. Because proper MHC I loading and recognition requires complexing with B2M, methods to deplete its expression abrogate the HvG effect and augment engraftment success rate. In conjunction with using cell types that employ MHC-independent recognition pathways, such as iNKT and NY-ESO TCRs as mentioned above, successful adoption of an allogeneic “off-the-shelf” cell therapy becomes feasible.

Engineered MHC I deficient cells may be employed to successfully target tumors through a combination of CRISPR-Cas 9 knockdown of endogenous TCR and transduction of a CAR moiety to provide MHC I-independent tumor recognition capacity. Using lentivirus transduction, Ren et al. produced allogeneic CD19-targeting CAR-T (CAR19-T) cells possessing abrogated expression of the TCR, HLA class I molecules, and immune checkpoint PD-1 (136). When CAR19-T cells were co-cultured with allogeneic PBMCs, alloreactivity was largely diminished indicating their favorable profile for allogeneic engraftment. This was similarly applicable within our studies BMCA-targeting CAR iNKT (BCAR-iNKT) cells that were depleted for B2M (11). Within an *in vivo* multiple myeloma mouse xenograft model, depleted BCAR-iNKT cells achieved longer survival than non-depleted counterparts while still maintaining total tumor clearance. For both CAR-T and CAR-iNKT based therapies, ablation of B2M or HLA I expression renders a potent strategy to abrogate HvG effects and promote long-term graft persistence.

Human Leukocyte Antigen-DR isotype (HLA-DR) is an MHC class II surface antigen-presenting molecule, with expression restricted to professional APCs, including B cells, macrophages, and dendritic cells. Morphologically, HLA-DR is a heterodimer composed of α and β chains then complex to recognize its cognate CD4 co-receptor (137). In contrast to MHC I which presents cytosolic antigens, MHC II molecules present exogenous antigens for recognition by single positive CD4 helper T cells (Figure 2B). Expression of HLA-DR mediates both direct and indirect HvG effect through respective recognition of either graft-derived or endogenous APCs (138). The direct pathway holds more relevance for acute allograft rejection, while the indirect pathway remains a causal factor for chronic graft rejection (139). Through depletion of HLA-DR expression on effector cell transplants, alloreactive host lymphocytes cannot be primed for both direct and indirect HvG effect.

As a relatively early technology, zinc finger nucleases (ZFNs) were successfully utilized to ablate HLA expression in allogeneic hematopoietic stem cells (HSCs) while maintaining pluripotency (140). More recently, CRISPR-Cas 9 multiplex gene editing has provided an avenue for more specific disruption of HLA II α genes, including HLA-DRA, DQA, and DPA (141). After 13 days of quadruple gRNAs transfection, Lee et al. observed that the gene-edited CD3^+^ T cells composed 62.1% of HLA-I/II-double-negative cells, while retaining surface phenotype and functionality. However, for generation of universally compatible iPSCs (142), results concluded that TALEN restriction enzymes had the greatest consistency; they were more maneuverable than ZFNs and had fewer off-target events than CRISPR/Cas 9. Even when stimulated with high concentrations of IFN-γ, TALEN-treated fibroblasts lacked HLA-DR expression, which was maintained within iPSCs generation (142). HLA-DR knockout remained effective and safe in these therapies, suggesting their potential to modify effector cells to prevent HvG effect in cancer immunotherapy (143).

Class II transactivator (CIITA) is a transcriptional coactivator that regulates IFN-γ-activated MHC gene expression. CIITA primarily controls expression and production of MHC class II molecules but also plays a role in supporting constitutive expression of MHC I expression (144). Thus, CIITA is a viable target to induce knockout of MHC II, thus reducing HvG incidence mediated by HLA class II molecules. Through multiplex gene editing, researchers disrupted the CIITA gene in human PSCs (145). *In vitro* and *in vivo* immunoassay suggested that alloreactive response against gene-edited PSCs was minimized, suggesting this strategy’s potential to develop a universal cell therapy (146). Our own experiments to develop allogeneic HSC-derived iNKT cells, have produced CIITA-KO and B2M-KO universal BCAR iNKT cells that could completely abrogate HvG effect (6), emphasizing effectiveness within fully differentiated T cell subsets.

### 4.2. Overexpression of inhibitory ligands

HLA class I histocompatibility antigen, alpha chain E (HLA-E) and alpha chain G (HLA-G) are non-classical MHC class I molecules that act as inhibitory ligands for NK cells (147). Recognition of HLA-E by CD94/NKG cell receptors, protects targeted cells from NK cell-mediated lysis (148). Although HLA ablation may abate targeting from host lymphocytes, these cell therapies alone remain vulnerable to NK cell recognition pathways (148, 149). Engineering HLA-E/HLA-G overexpression may provide protective effects within effector cells, thus safeguarding them from innate targeting to enhance *in vivo* persistence (150, 151). Zhao et al. successfully generated human embryonic stem cells (hESCs) with high expression of HLA-G (152), providing protective effects against NK cell-mediated cytotoxicity. In a similar vein, Torikai et al. utilized a Sleeping Beauty (CD19RCD28) transposon system to homogenously induce overexpression of HLA-E in CAR19-T cells, consequently reducing measured cell lysis *in vitro* (153). Using AAV-mediated gene editing to insert the gene for HLA-E at the B2M locus in hPSCs, Gornalusse et al. generated cells overexpressing HLA-E single-chain dimers or trimers while disrupting HLA-A/B/C production. These gene-edited human PSCs demonstrated strong resistance against NK cell killing as well as minimal recognition by CD8^+^ cytotoxic lymphocytes (154). Regardless of strategy, insertion of HLA-E/G genes provided sufficient protection against NK cell-mediated killing enhancing effector cell survival and persistence (Figure 2C).

CD47 is a transmembrane glycoprotein expressed universally among human cells (155, 156), and is upregulated within tumors to promote survival (157–159). Through interaction with signal receptor protein-alpha (SIRPα) expressed on macrophages, targeted cells inhibit phagocytosis and cell digestion to evade eradication (155, 156, 159). Macrophages largely contribute to HvG effect through both direct killing as well as their pro-inflammatory function in activating markers, cytokine profile, and engagement in ADCC (160–162). To prevent macrophage killing, exploitation of CD47/SIRPα pathway provides a potential remedy; akin to CD47^hi^ tumors, graft cells engineered to overexpress CD47 evade macrophage-mediated phagocytosis (163). By combining lentivirus transduction of CD47 with gRNA guided CRISPR-Cas 9 knockdown of B2M and CIITA, it is feasible to engineer HLA^−^CD47^hi^ effector cells that are immune to both T cell and macrophage mediated HvG effect (163, 164). Compared to non-engineered allogeneic grafts, CD47 modified cells showed enhanced survival and persistence within *in vivo* mouse xenograft models (163). By incorporating CD47 to evade macrophage killing, engrafted effector cells promote low HvG response, enhancing their long-term ability to suppress tumor growth (Figure 2D).

### 4.3. Disruption of CD52 to avoid total lymphodepletion

Lymphodepletion is a crucial step in cell therapies to reduce suppressive immune cells and decrease immunogenicity of the host immune system prior to cell transfer. One method for temporary lymphodepletion is to use alemtuzumab, a monoclonal antibody that targets CD52 and induces ADCC against mature lymphocytes but not naïve lymphocyte progenitors (165); however, this method cannot be used for adoptive transfer due to indiscriminate targeting of healthy and cancerous lymphocytes (165). One genetic engineering approach to address this challenge is the disruption of CD52 expression with TALEN mRNA to eliminate the binding cite of alemtuzumab in order to maintain antitumor efficacy. This approach is used in CD19-CAR-T cells against lymphoma, generating a CAR-T cell product capable of allogeneic engraftment (165). Engineering CD52 deficient CAR-T cells allows for concurrent treatment of alemtuzumab and CD19-CAR-T cells for combined host cell depletion and donor cell engraftment. Several clinical trials utilize this TALEN-mediated CD52-knockout for successful selective lymphodepletion in allogeneic CD19-CAR-T cell therapy (166). Genetically engineering a mechanism for selective lymphodepletion provides an opportune avenue for allogeneic transfer of CAR-T cells without the risk of GvHD.

## 5. Engineering strategy to improve safety profile

Despite the aforementioned engineering strategies to ameliorate GvHD, safety remains an issue for allogeneic cell products, which may cause unpredictable effects post therapeutic cell infusion (2, 3). Engineered cell products such as CAR-T cells might produce lifetime sustainment in patients as well as elicit adverse effects related to permanent gene transfer (2). CRS and neurotoxicity are also common safety risks for cell-based immunotherapy (3, 4). To achieve more desirable safety profile and avoid harm to patients, the introduction of suicide genes has provided an effective approach to allow selective destruction of modified cells at a designated time point (2, 5).

Suicide gene technology could be classified into three groups (5). First, gene-directed enzyme prodrug therapy (GDEPT) converts a nontoxic drug into a toxic compound through a genetically encoded molecule, usually the herpes simplex virus thymidine kinase (HSV-TK) (1, 2, 5). Second, apoptotic genes such as human caspase-9 could be linked to a drug binding domain for conditional dimerization (5), and administration of a non-therapeutic small molecule dimerizer would induce apoptosis (5). The third category utilizes monoclonal antibody-mediated cell removal mechanisms to eliminate allogeneic cells with a specific membrane protein expression (e.g., CD20) (5).

Among the three categories, HSV-TK is the most extensively tested suicide gene therapy utilized in human cells (2). The HSV-TK gene has been inserted into donor cells using retroviral or lentiviral transduction, which are then selected for purity (3, 5). The transduced cells constantly express the TK gene, which could phosphorylate pyrimidine and guanosine analogs (e.g., acyclovir, ACV; ganciclovir, GCV; penciclovir, PCV) (6). The phosphorylation leads to DNA misconformation through integration of DNA polymerase, eventually resulting in cell death (6). Thus, administration of guanosine analogs could eliminate more than 90% of circulating TK gene-engineered cells in most cases (5).

Our previous studies demonstrated the use of TK suicide gene in allogeneic cell-based cancer immunotherapy, where the lentivector was constructed through integration of an iNKT TCR gene with an sr39TK gene (3). The humanized SR39 (SR39h) was tested to be the most sensitive TK variant to suicide induction (6). Human HSCs were transduced with this Lenti/iNKT-sr39TK vector and further differentiated into allogeneic iNKT cells (3). The yield and functions of iNKT cell products were not affected by the incorporation of a suicide gene, displaying strong antitumor killing efficacy to multiple caners including melanoma, myelogenous leukemia, lung cancer, prostate cancer, and multiple myeloma (3). Therefore, TCR engineering, as well as CAR engineering and HLA ablation, could be combined with suicide gene engineering at the same time (3). Furthermore, Qin et al. established a safety-cell system through linking HSV-TK gene and a cell-division gene (CDK1), protecting the suicide system from inactivation in dividing cells (3). They found that the safety-cell system with GCV could eliminate all mitotically active cells and thus represented a safety measure before cell infusion into a patient (167). The incorporation of suicide genes provides additional safety measures for allogeneic cell-based immunotherapy, minimizing the potential risks for cancer patients for clinical development of future drugs.

## 6. Discussion

As a promising immunotherapy, allogeneic cell-based therapy has been increasingly studied for cancer treatment. This review comprehensively introduced current strategies of genetically engineering cells, especially allogeneic cell products, for strengthening the anti-tumor reactivity and reducing alloreactivity, thereby improving treatment efficacy. Cell engineering provides a platform to enhance anti-tumor efficacy of engrafted cells. Patient-derived T cells genetically engineered to express CAR are the typical base for CAR-T cell therapy but still carry a potential risk for GvHD and HvG. In response to this problem, alternative cells, such as NK and unconventional innate T cells, are utilized for safer CAR engineering. Another engineering method is the incorporation of cytokine armoring genes, which leads to the production of cytokines required for T cell growth, including IL-15, IL-2, IL-7, IL-12, and IL-21, hence strengthening tumor-killing abilities. Moreover, the blockade of several immune checkpoints, such as PD-1, CTLA-4, and LAG-3, represents an alternative way of inducing anti-tumor immunity. Eliminating these crucial ligand-receptor interactions prevents tumor cells from bypassing eradication from the immune system, as these greatly confer immune resistance to cancerous cells. Additionally, immune checkpoint blockade therapies have been utilized in combination with CAR T cell therapy. One study has shown that for human triple-negative breast cancer cells with mesothelin overexpression that can be targeted by CAR T cells, by counteracting the inhibitory effect of PD-1 on CAR-T cells through CRISPR-Cas9 ribonucleoprotein-mediated editing, PD-1^hi^ population was significantly reduced with a minimal impact on the proliferation of T cells. In contrast, CAR-T cells with PD-1 disruption demonstrated improved tumor control and relapse prevention compared to CAR T cells with or without αPD-1 antibody blockade. These findings support the integration of immune checkpoint blockade with CAR-T cells to advantageously manipulate solid tumors and offer an alternative method to CAR-T cell therapy (168). Graft anti-tumor responses can also be improved through the modulation of transcription factors and epigenetic enzymes, two widely studied methods being the knockdown of EZH1 and DNTM3A. In addition, maintaining the cytotoxicity of NK cells through the induction of CD16 has shown promise in suppressing tumor cells as well.

To ensure the safety of allogeneic cell-based therapy, strategies have been designed to reduce post-treatment alloreactivity. One way to ameliorate potential GvHD is to ablate TCR expression, which can be achieved through disruption of endogenous TCR and substitution with a CAR moiety to maintain tumor recognition. Alternatively, incorporating the NY-ESO-1 TCR transgene enables the encoding of a non-conventional TCR, which is not implicated in alloreactive pathways. In addition, unconventional T cell subsets, including iNKT cells, provide possibilities of limiting alloreactivity through endogenous, HLA-independent recognition pathways. From the perspective of the HvG effect, studies have indicated that ablating HLAs, including B2M, HLA-DR, and CIITA could successfully augment graft success rate. Moreover, overexpressing inhibitory ligands, such as HLA-E, protects effector cells from NK cell-mediated killing, whereas CD47 incorporation enables effector cells to evade macrophages activity. Finally, disrupting the expression of CD52 plays a vital role in successful lymphodepletion, an essential step for preventing GvHD. The combination of various strategies for enhancing anti-tumor efficacy and suppressing alloreactivity suggests a promising future for allogeneic cell products in cancer immunotherapy.

One type of current allogeneic CAR cell therapy, ALLO-715, utilizing BCMA-targeting CAR T cells, has been tested in part A of in-human phase 1 trial against multiple myeloma (MM) named UNIVERSAL (169). TALEN technology enables two additional alterations in this therapy: TRAC knockout and CD52 knockout. Removing TRAC downregulates the expression of the TCRαβ complex, thereby preventing the recognition of histocompatibility antigens mediated by TCRαβ, lowering the risk of GvHD consequently. In addition, CD52 is expressed on multiple immune cell types whose implications with HvG effect would diminish ALLO-715 persistence. Therefore, to ensure the cell expansion and persistence of ALLO-715, patients undergo lymphodepletion in conjunction with ALLO-647 engraftment, using anti-CD52 blockade. Favorable outcomes, such as lower rates of CRS and neurotoxicity, reflect the feasibility and overall safety of ALLO-715 administration.

Another existing type of off-the-shelf allogeneic CAR-T cells is UCART19, whose mRNA encoding TALENs knocks out the TCR-α-constant-chain-encoding and CD52 genes (12). Such a function reduces the risk of GvHD in utilizing allogeneic UCART19. Therapeutic effects of UCART19 have been tested in two clinical trials, PALL trial for children and CALM trial for adults, for treating pediatric and adult B-cell acute lymphoblastic leukemia, where UCART19 cells generated through recombinant lentiviral transduction were infused after the 7-day lymphodepletion treatment. Despite the occurrence of adverse events, such as CRS, satisfying outcomes include *in vivo* expansion of UCART19 and antileukemic response in a relatively safer manner. These trials demonstrate the feasibility of employing allogeneic UCART19 to treat B-cell acute lymphoblastic leukemia, especially when facing the rapid progression of the disease and the unavailability of autologous CAR-T-cell treatment. In addition, such trials provide a template for practically incorporating allogeneic cell products with multiple genetic engineering strategies, heralding the promise of allogeneic cell-based cancer therapy. In spite of the prospect of allogeneic CAR-T-cell products like UCART19, one accompanying limitation is manufacturing complexity (6). Since the risk of GvHD resides in HLA incompatibility for conventional TCRαβ T cells, endogenous TCR expression attenuation is required, which guarantees safety but simultaneously increases production difficulty.

In response to this problem, generating stem cell-derived products to express CARs and TCRs for tumor suppression offer possible alternatives (13). In addition to conventional CAR-T cells, CAR-engineered stem cells like CAR-NK and CAR-myeloid cells lead to a more extensive anti-tumor response. One study has demonstrated the performance of CAR-NK cell therapy against CD19-positive lymphoid tumors (170). Researchers derived the HLA-mismatched anti-CD19 CAR-NK cells from cord blood. Retroviral vector for transduction encoded anti-CD19 CAR, IL-15, and inducible caspase 9. In phase 1 and 2 trials, most patients experiencing engineered CAR-NK cell infusion showed quick responses to the therapy without accompanying side effects such as CRS, neurotoxicity, and hemophagocytic lymphohistiocytosis, except for hematologic toxic events related to lymphodepletion chemotherapy. In addition, GvHD did not occur after CAR-NK cell administration, indicating the viability and safety of allogeneic CAR-NK cell therapy.

Despite the promising features of current allogeneic CAR cell therapies mentioned above, a few drawbacks should be considered. In the application of BCMA-targeting CAR-T cells and UCART19 cells, the ablation of TCR and CD52 might lead to increased manufacturing difficulties and high costs (6, 12, 118, 171, 172). In contrast, although the cord blood-derived CAR-NK cells effectively avoid the risk of GvHD, the sensitive and complex process of thawing blood units could potentially lower the treatment’s efficacy. Furthermore, the availability of allogeneic CAR cell therapies depends on several factors. For example, due to the limited length of lentiviral/retroviral vectors required for transduction (169), transgene should be optimized before practical administration. Development of new gene delivery systems might overcome this problem. Although the occurrence of side effects is controlled at a relatively low level, such adverse problems remain unsolved. As seen in CRS, since IL-6, IL-1, and nitric oxide (NO) secreted by recipient macrophages mediate the severity of the syndrome rather than cytokines obtained from CAR T cells, blocking IL-6 and IL-1 and inhibiting inducible NO synthase (iNOS) provides an ameliorating effect (173). Moreover, as human microglia generate iNOS and proinflammatory cytokines *via* IL-1 activation, IL-1 blockade also plays an essential role in alleviating CAR-T cell-mediated neurotoxicity (173). Further studies should focus on discovering alternatives for improving the safety control of allogeneic CAR cell therapies.

Similar to the UCART19 therapy mentioned above, genetically engineered stem cells can also be utilized in allogeneic treatment. Specifically, in HSC transplantation (HSCT), it is possible to produce immune cells that target specific tumors over an extended period using genetically modified cells. On the contrary, PSCs, including ESCs and iPSCs, can self-renew while preserving pluripotency and provide endless supplies for target cells. Furthermore, the accessible source PBMCs allows for convenient iPSC reprogramming. By starting the engineering process with a small number of stem cells, the cost can be controlled while efficiency is enhanced.

However, while various engineering strategies and high accessibility of cell sources indicate the potential application of stem cell-based cancer therapy, several limitations are worth mentioning. For example, incorporating additional genes such as the sr39TK/GCV suicide switch may increase the immunogenicity and dependence on the cell cycle (6). Moreover, during the induction of T cells from iPSC and T-iPSC, uncharacterized serum and feeder cells of murine origin occur despite the successful generation, which is incompatible with the production at a clinical level (19). Therefore, future investigations should focus on improving the overall quality of genetically engineered cells and optimizing the generation process, thereby further improving the practicality of deriving immune cells from undifferentiated stem cells and advancing the development of allogeneic cell-based immunotherapy for cancer.

## Author contributions

Y-RL and LY: conceptualization. YC, AK, YZ, YF, and MW: writing-original draft preparation. YC, AK, MW, YF, and Y-RL: writing-review and editing. YC and YF: visualization. Y-RL and LY: supervision. All authors have read and agreed to the published version of the manuscript.

## Funding

This work was supported by an Ablon Scholars Award (to LY), a UCLA BSCRC Innovation Award (to LY), and an M. John Pickett Post-Doctoral Fellow Award (to Y-RL).

## Conflict of interest

Y-RL and LY are inventors on patents relating to this article filed by UCLA. LY is a scientific advisor to AlzChem and Amberstone Biosciences, and a co-founder, stockholder, and advisory board member of Appia Bio.

The remaining authors declare that the research was conducted in the absence of any commercial or financial relationships that could be construed as a potential conflict of interest.

## Publisher’s note

All claims expressed in this article are solely those of the authors and do not necessarily represent those of their affiliated organizations, or those of the publisher, the editors and the reviewers. Any product that may be evaluated in this article, or claim that may be made by its manufacturer, is not guaranteed or endorsed by the publisher.
